# Electroencephalography-based neurofeedback as treatment for post-traumatic stress disorder: A systematic review and meta-analysis

**DOI:** 10.1192/j.eurpsy.2019.7

**Published:** 2020-01-31

**Authors:** Steinn Steingrimsson, Gorana Bilonic, Ann-Catrin Ekelund, Tomas Larson, Ida Stadig, Mikael Svensson, Iris Sarajlic Vukovic, Constanze Wartenberg, Olof Wrede, Susanne Bernhardsson

**Affiliations:** 1 Region Västra Götaland, Psykiatri Affektiva, Department of Psychiatry, Sahlgrenska University Hospital, Gothenburg, Sweden; 2 University of Gothenburg, Sahlgrenska Academy, Institute of Neuroscience and Physiology, Gothenburg, Sweden; 3 Region Västra Götaland, Medical Library, Skaraborg Hospital, Lidköping, Sweden; 4 Region Västra Götaland, Medical Library, Sahlgrenska University Hospital, Gothenburg, Sweden; 5 Region Västra Götaland, HTA-Centrum, Gothenburg, Sweden; 6 Region Västra Götaland, Crisis and Trauma Unit, Gothenburg, Sweden; 7 Research and Development Primary Health Care, Gothenburg, Sweden

**Keywords:** Biofeedback, EEG, neurofeedback, post-traumatic stress disorder, systematic review

## Abstract

**Background.:**

Post-traumatic stress disorder (PTSD) is debilitating for patients and society. There are a number of treatment methods albeit not all patients respond to these and an interesting method using electroencephalography-based neurofeedback (EEG-NF) has become more prominent in recent years. This systematic review aimed to assess whether EEG-NF, compared with sham NF, other treatment, or no treatment, is effective for PTSD. Primary outcomes were self-harm, PTSD symptoms, level of functioning and health-related quality of life.

**Methods.:**

Systematic literature searches for randomized controlled trials (RCTs) were conducted in six databases. Random effects meta-analysis was performed. Certainty of evidence was assessed using the Grading of Recommendations Assessment, Development, and Evaluation.

**Results.:**

Four RCTs were included (123 participants). Suicidal thoughts were significantly reduced after EEG-NF compared with a waiting list in a small study. PTSD symptoms were assessed in all studies with different instruments. Results were consistently in favor of EEG-NF with large effect sizes (standardized mean difference −2.30 (95% confidence interval: −4.37 to −0.24). One study reported significantly improved level of executive functioning and one study a reduction in use of psychotropic medication. Complications were scarcely reported. Certainty of evidence was assessed as very low for the four assessed outcomes.

**Conclusions.:**

Based on four RCTs, with several study limitations and imprecision, it is uncertain whether EEG-NF reduces suicidal thoughts, PTSD symptoms, medication use, or improves function. Although all studies showed promising results, further studies are needed to increase the certainty of evidence.

## Introduction

Post-traumatic stress disorder (PTSD) has been defined as the development of characteristic symptoms following exposure to extreme traumatic stressor(s) including persistently re-experiencing the trauma leading to negative changes in cognition and mood, and avoidance behavior [1]. This DSM-5 definition includes a time frame of at least 1 month with clinically significant distress or impairment in social, occupational, or other important areas of functioning. Another way of describing PTSD is as a composite of “somatic, cognitive, affective, and behavioral effects of psychological trauma” [2] that often leads to problems in maintaining an occupation, establishing social support, and increased rate of disability support [3]. Common PTSD symptoms include re-experiencing of trauma (flashbacks), sleep disturbance, irritability, and feelings of guilt.

Furthermore, a number of somatic diseases are associated with PTSD. In a 3-year follow-up study of European refugees after the war in Bosnia and Herzegovina, the occurrence of medical conditions, such as high blood pressure (38%) and heart disease (31%), were frequent among those diagnosed with PTSD and depression [4]. As a reference, global prevalence of raised blood pressure has been reported as 24% in men and 20% in women [5]. Furthermore, a population-based study showed increased risk for angina, heart failure, bronchitis, asthma, liver, and peripheral arterial disease in those with a history of trauma compared with a general population [6]. A more immediate threat to life is the increased risk for suicide in patients with PTSD. This risk was estimated to be around 10 times higher than in the general population in a registry study in Denmark, and when adjusted for other psychiatric comorbidities the risk was still 5.3 times higher in patients with PTSD [7].

The average prevalence of PTSD in upper-middle income and lower-middle income countries has been reported as 2.3 and 2.1%, respectively [8]. In national samples of the general adult population in the United States, Canada, and Sweden, lifetime prevalence is reported to be between 5.6 and 9.2% [9-12].

A particular problem in diagnosing PTSD may be the overlap with other psychiatric disorders. The National Comorbidity Survey data, a large survey of mental health in the United States, suggest that 16% of PTSD patients have one coexisting psychiatric disorder, 17% two, and 50% have three or more [13]. Most common comorbidities are depressive disorder, anxiety disorder, and substance abuse. Further difficulties in defining PTSD are both the definition of trauma and how populations are differently exposed to war or other external factors that increase the risk of developing symptoms of PTSD.

Today, healthcare providers around the world offer a noteworthy breadth of treatment methods for PTSD, most of which are based on evidence from intervention studies [14-18]. The most widely accepted methods are psychotherapy (e.g., cognitive behavioral therapy, exposure therapy, eye movement desensitization and reprocessing [EMDR]), and pharmacotherapy (especially selective serotonin re-uptake inhibitors) as well as a combination of these treatments. Moreover, social support and PTSD-adjusted physiotherapy are standard options in several countries—as complementary or therapies in their own right. Across all the mentioned alternatives, treatment lengths range from 8 weeks to several years. Specific treatments, with a rationale manual, often apply a 20–40 session model (approximately 20–40 weeks). Typically, open treatments (i.e., without manual) allow for a much longer time span than structured approach therapies. Despite this range of treatment options, there is not a complete success in recovery and new treatment options need to be explored in order to provide treatment resistant patients with alternatives.

Neurofeedback (NF) is a noninvasive treatment method for re-establishing the electrophysiological activity of the brain, with the aim of reducing symptoms related to over- or under-arousal within different parts of the brain [19]. It is believed that NF, via endogenous neuromodulation (in contrast to exogenous methods such as transcranial magnetic stimulation or other electrical stimulations to the skin), can change neuronal activity or connectivity and thus indirectly modify a person’s behavior [20]. Using the older method based on operant conditioning, brainwave activity is measured using EEG and fed back to the person via a simple stimulus (visual or auditory), which enables the brain to sustain the desired activity [21]. The development of NF can be traced back to the 1960s when electroencephalography (EEG) patterns were associated with behavior, and in the 1970s, the method was tested among patients with Attention Deficit Hyperactivity Disorder (ADHD) and epilepsy [19]. In recent years, the use of infralow frequencies recorded on EEG has gained more attention, which was developed based on findings in functional magnetic resonance imaging whereby the external stimulus is not aimed at operant conditioning but rather that low (<0.1 Hz) frequencies guide the feedback in a nonrewarding/nonpunishing stimulus feedback [22].

Positive effects of NF have been described on symptoms of ADHD [23] and depression [24], while the evidence base for EEG-based NF as treatment of PTSD is limited. The first published trial dates to 1991, when the method was reported to show beneficial effects among United States war veterans with combat-related PTSD [25]. Prior to this, the method had been found promising to treat alcohol us disorder in war veterans; an alcohol-related problem that was likely due to PTSD but not diagnosed as such [26]. Since then, several trials have been conducted, showing tentative positive effects. Two reviews have been published that summarized the studies [27, 28], concluding that study results were promising but that evidence is limited for EEG-based NF. However, these reviews did neither perform any meta-analyses nor did they assess the certainty of the evidence systematically and transparently since their publication additional trials have been conducted. Therefore, there is a need to synthesize the results of these trials and to assess the evidence base for the treatment method in view of implementing it in clinical practice, as well as to determine further research needs.

The aim of this systematic review was to evaluate the effects of EEG-based NF in patients with PTSD compared with sham NF, other interventions, or no intervention. Primary outcomes were self-harm, PTSD symptoms, level of functioning, and health-related quality of life, and secondary outcomes were sick leave, medication use, and complications.

## Methods

The systematic review was conducted as part of a health technology assessment (HTA) performed at HTA-centrum, Sahlgrenska University Hospital in Gothenburg, Sweden [29]. A focused research question based on the literature search framework: Population, Intervention, Comparison, Outcome (PICO) and eligibility criteria were developed before the searches were performed. The review is reported according to the Preferred Reporting Items for Systematic Review and Meta-Analysis statement [30]. The review was not registered.

### Eligibility criteria

The articles eligible for inclusion had to fulfill all selection criteria: (i) study participants were adult (≥18 years) and diagnosed with PTSD, (ii) the NF intervention was based on electroencephalogram (EEG), and (iii) the intervention was compared to sham NF (i.e., simulated), other treatment (e.g., psychotherapy, medication, physiotherapy, EMDR), or no treatment.

Primary outcomes were self-harm (including suicidality and suicidal thoughts), PTSD symptoms, level of functioning, and health-related quality of life. Secondary outcomes were sick leave, medication use, patients’ experiences of the treatment, and complications.

Randomized controlled trials (RCTs), cohort studies, case series with ≥10 subjects (for analysis of complications), qualitative studies, and cost/economic studies were eligible for inclusion. Studies had to be in English or Scandinavian languages (Danish, Norwegian, or Swedish). No restriction was applied to date of publication. Systematic reviews published since 2016 were included in the search for purposes of probing reference lists for identification of other eligible studies, but not included in the analyses of treatment outcomes.

### Patient involvement

The PICO was reviewed by a patient with PTSD currently undergoing treatment at one of the author’s clinical workplace. This person confirmed that the outcomes at issue and their priority were relevant.

### Data sources and study selection

During November 2018, two authors (I.S., A.-C.E.) performed a systematic search in PubMed, Embase, the Cochrane Library, Cinahl, PsycINFO, Web of Science, and a number of HTA databases. ClinicalTrials.gov was searched for relevant completed and ongoing trials. All databases were searched from inception to November 16, 2018. In PubMed, the following search string was used: ((Neurofeedback[mh]) OR (neurofeedback[tiab] OR neuro-feedback[tiab]) OR ((brainwave[tiab] OR alpha[tiab] OR electromyography[tiab] OR electromyographic[tiab] OR EEG[tiab] OR electroencephalography[tiab] OR electroencephalographic[tiab]) AND (biofeedback*[tiab] OR feedback*[tiab] OR bio-feedback*[tiab]))) AND ((Stress Disorders, Post-Traumatic[mh]) OR (PTSD[tiab] OR ((post-traumatic[tiab] OR post-traumatic[tiab]) AND (stress[tiab] OR neuroses[tiab] OR neurosis[tiab] OR disorder*[tiab])))) with no search limits. The search strings used in Embase, the Cochrane Library, Cinahl, PsycINFO, Web of Science, HTA databases, and ClinicalTrials.gov are analogue to that described above.

Reference lists of relevant articles were scrutinized for additional references. These authors conducted the literature searches, selected studies, and independently of one another assessed the obtained abstracts and made a first selection of full-text articles for inclusion or exclusion. Any disagreements were resolved in consensus. The remaining articles were sent to all authors. All authors read the articles independently of one another and decided in a consensus meeting which articles should be included in the review. Excluded studies and reasons for exclusion are presented in Supplementary Table S1.

### Data collection process

One author (S.S.) extracted data on study characteristics and outcomes and another author (C.W. or S.B.) verified the data extraction. Data were extracted on study design, follow-up, intervention components, control intervention components, population demographics (including age and gender), outcomes, outcome measures, and main findings.

### Assessment of risk of bias and certainty of evidence

Risk of bias was assessed at study level and certainty of evidence at outcome level. All authors critically appraised the included studies using a checklist for assessment of RCTs from the Swedish Agency for Health Technology Assessment and Assessment of Social Services [31]. This checklist is based on the Cochrane risk of bias tool [32] and assesses selection bias, performance bias, detection bias, attrition bias, reporting bias, and conflicts of interest. Any disagreements were resolved by discussion among all authors.

The Grading of Recommendations Assessment, Development and Evaluation (GRADE) approach was used to assess overall certainty of evidence [33]. All factors pertaining to five categories were assessed: study limitations/risk of bias (including randomization, blinding, follow-up, dropouts, compliance, and intention-to-treat analysis); consistency (including direction and magnitude of effect across studies and overlap of confidence intervals [CIs]); directness (including setting, population, intervention, control, outcome, and comparison—in other words, the generalizability); and precision (including sample size and width of CIs). To assess publication bias, “ClinicalTrials.gov” was searched. Relevant studies identified that were listed as completed, but had not been published, were examined. Because all included studies were RCTs, we initially assigned a high certainty level, but rated down one or more levels to moderate, low, or very low if issues with risk of bias, consistency, or other GRADE criteria were detected.

### Data synthesis and analysis

The results of each study were summarized and risk of bias was assessed per outcome. When possible, data were combined in meta-analysis for investigation of the aggregated effect. Because different scales were used, we calculated standardized mean differences (SMD) and their 95% CIs. Statistical heterogeneity was assessed with the *χ* ^2^ and *I* ^2^ statistics. Because heterogeneity was present (*I* ^2^ > 30%), we used a random-effect model. The meta-analysis was performed in Revman 5.3 (The Nordic Cochrane Centre, The Cochrane Collaboration, Copenhagen, Denmark).

## Results

### Search results

**Figure 1.**
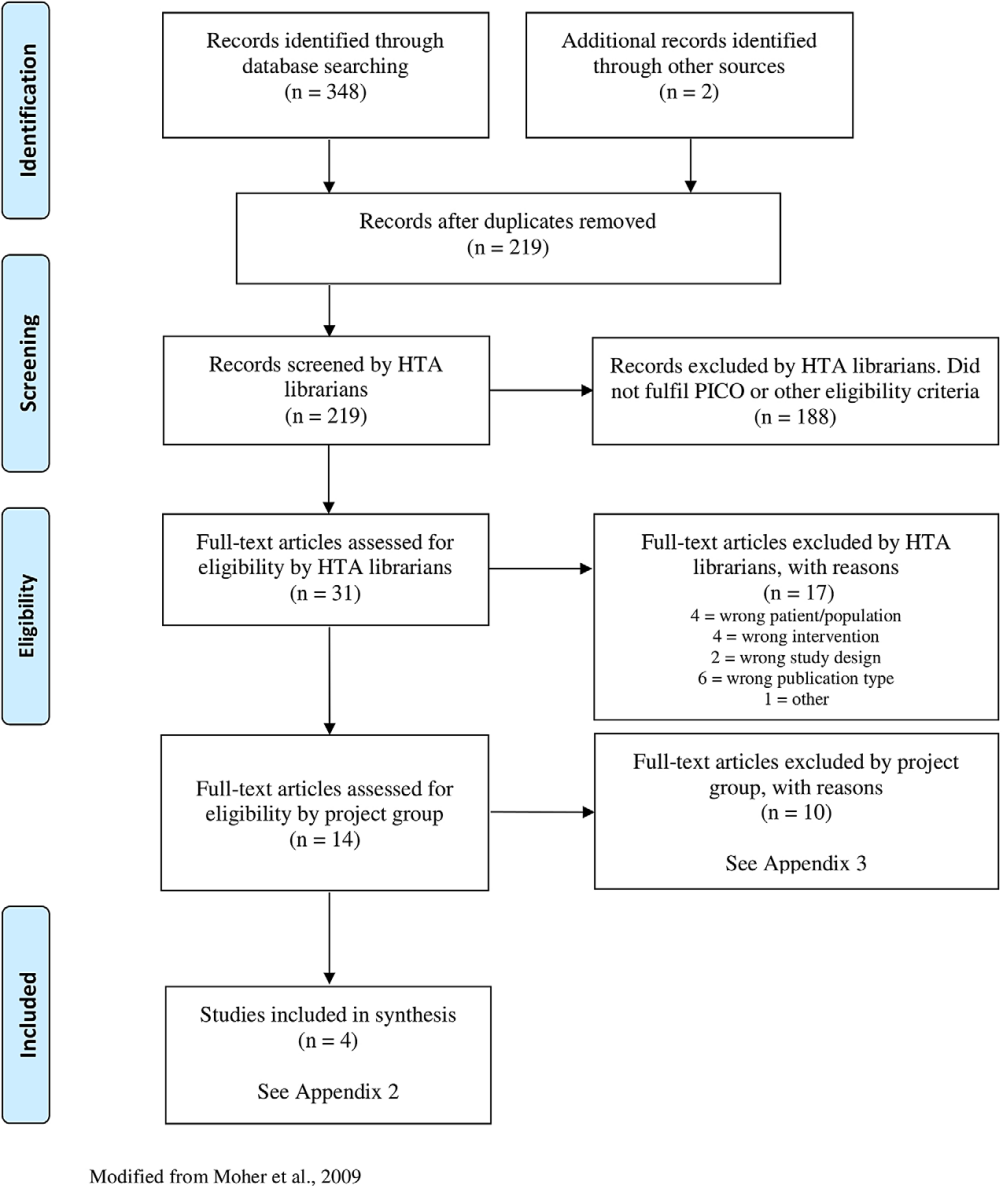
Flow diagram of selection process.

The literature search identified 219 records after removal of duplicates. After reading the abstracts, 188 articles were excluded. Another 17 articles were excluded by two authors (A.-C.E. and I.S.) in consensus after reading the articles in full text. The remaining 14 articles were sent to all authors, and four publications, reporting four RCTs, were finally included. A flowchart of the study selection process is presented in Figure 1. The search in ClinicalTrials.gov identified 17 relevant trials. Of those, one trial (ClinicalTrials.gov identifier: NCT01591408), designed to compare NF with sham-NF, was completed in 2016. However, no publication of the study could be found.

### Characteristics of included studies

Four randomized controlled studies, involving a total of 123 patients (68% male), met the inclusion criteria and were included in the review. The studies were conducted between 1991 and 2016; three in the United States and one in Iran. Of the four studies, three [34–36] compared EEG-based NF treatment with no intervention (waiting list), and one [25] with standard treatment. All four studies measured self-reported PTSD symptoms, using various scales. One study each reported self-harm [34], level of functioning [35], and medication use [25], as further described below. Treatment period ranged from 4 to 12 weeks. Only one study [25] reported long-term effects, measured at 30 months post-treatment.

All studies were small (*n* = 10–52) and were assessed as having moderate to high risk of bias, some indirectness, and imprecision. Problems in the included studies were identified with confounding factors, self-reported outcomes with no blinding, and lack of published protocols. The included studies, their design, and patient characteristics are presented in Table 1. Assessment of risk of bias is presented in the outcome tables and a summary of key findings is presented in Table 2.

**Table 1.** Characteristics of included studies

First author, year, country	Study design	Length of follow-up	Study groups; intervention vs. control	Patients (*n*)	Mean age (years)	Men (%)	Outcome variables
Kelson, 2013, U.S. [34]	RCT	Final assessment at end of 4 weeks treatment	I: EEG-based neurofeedback: 20 sessions, 5 times/week during 4 weeks C: waiting list (offered EEG-based neurofeedback at end of study)	Veterans with PTSD diagnosis I: 7 C: 5	I: 53.8 C: 49.8	100	PTSD symptoms, measured with self-constructed scale based on symptoms included in diagnostic criteria Suicidal thoughts, measured in one item in PTSD symptom scale
Noohi, 2017, Iran [35]	RCT	Final assessment at end of 45 days treatment	I: EEG-based neurofeedback: 25 sessions, 4 times/week during 45 days C: no intervention (?)	Patients with PTSD I: 15 C: 15	25–60 (mean not reported)	100	PTSD symptoms, measured with the impact of event scale-revised (IES-R) Level of functioning, measured with cognitive performance tests
Peniston, 1991, U.S.[25]	RCT	Thirty months after treatment	I: EEG-based neurofeedback: 30 sessions, 5 times/week C: standard treatment (traditional medical control, i.e., psychotropic medication and individual and group therapy) In both groups, a reduction of the initial psychotropic medication dosage was attempted	Veterans with chronic PTSD I: 15 C: 14	I: 36.1 (SD 2.6) C: 37.2 (SD 2.8)	100	PTSD symptoms, measured with the Minnesota Multiphasic Personality Inventory (MMPI) PTSD scale Psychotropic medication dosage
van der Kolk, 2016, U.S.[36]	RCT	Four weeks after end of 12 weeks treatment	I: initial pretraining in temperature biofeedback followed by EEG-based neurofeedback: 24 sessions 2 times/week for 12 weeks C: waiting list Both groups continued ongoing treatment (medication and psychotherapy)	Adults with treatment nonresponsive PTSD I: 28 C: 24	I: 46.0 (SD 12.9) C: 42.4 (SD 13.5)	24	PTSD symptoms, measured with the clinician administered PTSD Scale (CAPS) and the Davidson Trauma Scale (DTS)

Abbreviations: C, control; I, intervention; PTSD, post-traumatic stress disorder; RCT, randomized-controlled trial.

**Table 2.** Summary of findings, by comparison

Outcome	Number and type of studies (participants)	Absolute effect estimates	Certainty of evidence GRADE^a^
*EEG-based neurofeedback vs. sham neurofeedback*			
This comparison was not evaluated in any of the included studies.			
*EEG-based neurofeedback vs. other treatment*			
PTSD symptoms	1 RCT (*n* = 29)	Between-group difference in mean change pre-to-post-treatment: Δ 20.4 in favor of neurofeedback (scale 0–49), *p* < 0.05	⊕⃝⃝⃝^b^
Medication use	1 RCT (*n* = 29)	Number of patients with decreased medication use Decrease: 14/14 vs. 1/13 Between-group difference: *χ* ^2^ = 23.26, *p* < 0.05	⊕⃝⃝⃝^b^
*EEG-based neurofeedback vs. no treatment*			
Suicidal thoughts	1 RCT (*n* = 10)	Between-group difference in mean change pre-to-post-treatment: Δ 1.4 in favor of neurofeedback (scale 1–5), *p* = 0.002	⊕⃝⃝⃝^c^
PTSD symptoms	3 RCTs (*n* = 92)	Standardized mean difference at end of treatment −2.30 (95% CI −4.27 to −0.24), *p* = 0.03	⊕⃝⃝⃝^d^
Level of functioning	1 RCT (*n* = 30)	Wisconsin Card Sorting Test No. of errors Between-group difference in mean change pre-to-post-treatment: Δ 29.4 in favor of neurofeedback, *p* < 0.001 Tower of London Between-group difference in mean change pre-to-post-treatment: Δ 6.1 in favor of neurofeedback, *p* < 0.001	⊕⃝⃝⃝^e^
High certainty ⊕⊕⊕⊕	We are very confident that the true effect lies close to that of the estimate of the effect.
Moderate certainty ⊕⊕⊕⃝	We are moderately confident in the effect estimate: the true effect is likely to be close to the estimate of the effect, but there is a possibility that it is substantially different.
Low certainty ⊕⊕⃝⃝	Confidence in the effect estimate is limited: the true effect may be substantially different from the estimate of the effect.
Very low certainty ⊕⃝⃝⃝	We have very little confidence in the effect estimate: the true effect is likely to be substantially different from the estimate of effect.

a Certainty of evidence.

b Downgraded three steps for serious study limitations, indirectness, and serious imprecision (e.g., unclear randomization, lack of blinding, unclear whether data analyses were predefined, different preconditions in control treatment, one small study).

c Downgraded three steps for very serious study limitations, indirectness, and serious imprecision (e.g., self-reported outcomes with no blinding, unclear whether data analyses were pre-defined, different preconditions in control treatment, one very small study).

d Downgraded three steps for very serious study limitations and serious imprecision (e.g., different preconditions in control treatment, limitations in blinding, questions whether data analyses were predefined, heterogeneity).

e Downgraded three steps for very serious study limitations and serious imprecision (e.g., different preconditions in control treatment, unclear randomization, lack of blinding, questions whether data analyses were predefined).

### Outcomes

#### Self-harm

Self-rated suicidality was reported in one very small, unpublished RCT [34], with serious study limitations, indirectness, and imprecision. The study compared NF with a waiting list and measured self-rated suicidality on a scale from 1 to 5 (1 being the lowest possible answer; no suicidal thoughts) that was part of a nonvalidated questionnaire. The mean pretreatment rating was 2.2 (SD 0.8) in the NF group versus 1.0 (SD 0.0) in the control group. After 4 weeks treatment, the rating was 1.0 (SD 0.0) in the NF group versus 1.2 (SD 0.45) in controls. The difference in mean pretreatment to post-treatment change was 1.4 in favor of NF (*p* = 0.002). Based on our GRADE assessment (Table 2), we conclude that it is uncertain whether EEG-based NF compared with waiting list reduces self-rated suicidality in adult patients with PTSD (very low certainty of evidence).

#### PTSD symptoms

Effects on PTSD symptoms were assessed in all four included studies and are reported in Table 3. None of the studies compared NF with sham NF. One study [25] compared NF with standard treatment, comprised of traditional medical control, that is, psychotropic medication, and individual and group therapy. The study was assessed as having serious study limitations, indirectness, and serious imprecision. PTSD symptoms were measured by the Minnesota Multiphasic Personality Inventory PTSD Scale. Symptom data were collected at baseline, end of treatment and 30 months after treatment. The intervention group showed a larger reduction in symptoms post-treatment (between-group difference in change: 20.4 points, *p* < 0.05). At 30 months’ follow-up, relapse was reported in significantly fewer patients in the intervention group (3/15) than in the control group (14/14; *p* < 0.05).

**Table 3.** Reported effects on PTSD symptoms

Study	Number of patients (*n*)	Withdrawal or dropout	Results		Comments	Directness^a^	Limitations^a^	Precision^a^
			EEG neurofeedback	Waiting list or standard treatment				
Kelson, 2013 [34]	I: 7 C: 5	I: 2	PTSD symptoms questionnaire Pre: 72.8 (SD 12.6) Post (week 4): 44.2 (SD 9.6) Δ −28.6	PTSD symptoms questionnaire Pre: 69.4 (SD 10.0) Post (week 4): 78.8 (SD 9.0) Δ +9.4 Between-group difference in pre-to-post change: Δ −38.0, *p* < 0.01	Twenty-three-item instrument ranging from 23 to 115, lower values indicate fewer symptoms. NB: The PTSD symptoms questionnaire is not a validated instrument.	−	?	−
Noohi, 2017 [35]	I: 15 C: 15	0?	IES-R Pre: 47.2 (SD 7.6) Post (45 days): 30.4 (SD 6.2) Δ −16.8	IES-R Pre: 51.1 (SD 5.4) Post (45 days): 51.1 (SD 6.2) Δ 0.0 Between-group difference in pre-to-post change: Δ −16.8, *p* < 0.001	Number of withdrawals unclear. Twenty-two-item instrument, ranging from 0 to 88, lower values indicate fewer symptoms.	+	−	?
Peniston, 1991 [25]	I: 15 C: 14	0	MMPI PTSD Pre: 30.6 (SD 9.1) Post (28 days): 10.5 (SD 6.2) Δ −20.1	MMPI PTSD Pre: 35.9 (SD 7.2) Post (28 days): 36.2 (SD 5.3) Δ +0.3 Between-group difference in pre-to-post change: Δ −20.4, *p* < 0.05	MMPI PTSD is a scale based on 49 items related to PTSD that are part of a larger number of personality measures. The range of the scale is 0–49, lower values indicate fewer symptoms. NB: numbers for MMPI PTSD measured on a graph (not reported in article)	?	?	?
			PTSD relapse after 30 months Relapse: 3/15 (20%)	PTSD relapse after 30 months Relapse: 14/14 (100%) Between-group difference in pre-to-post change: −80%, *p* < 0.05				
van der Kolk, 2016 [36]	I: 28 C: 24	I: 6 C: 2	DTS Pre: 67.3 (SD 25.0) Post: 44.2 (SD 19.2) Δ −23.1 (−34.3%) 1 month follow-up: 36.5 (SD 19.3) Δ −30.8	DTS Pre: 63.0 (SD 18.2) Post: 58.2 (SD 20.6) Δ −4.8 (−7.6%) Between-group difference in pre-to-post change: Δ −13.4, *p* < 0.001 1 month follow-up: 65.5 (SD 20.3) Δ +2.5 Between-group difference in pre-to-1 month post change: Δ −33.3, *p* < 0.001	DTS ranges from 0 to 136, lower values indicate fewer symptoms. 95% CIs reported in the paper converted to SD.	+	+	+
			CAPS Pre: 79.5 (SD 16.9) Post: 43.0 (SD 20.2) Δ −36.5 (−45.9%)	CAPS Pre: 76.2 (SD 16.9) Post: 66.5 (SD 20.6) Δ −9.7 (−12.7%) Between-group difference in pre-to-post change: Δ −26.8, *p* < 0.001	CAPS ranges from 0 to 136, lower values indicate fewer symptoms. A score under 45 is considered as not meeting criteria for PTSD. A 20-point change in CAPS criteria indicates a clinically significant change. 95% CIs reported in the paper converted to SD.			
			1 month follow-up: 39.1 (SD 20.0) Δ −40.4 (−50.8%)	1 month follow-up: 65.5 (SD 20.3) Δ −10.8 (−14.2%) Between-group difference in pre-to-1 month post change: Δ −29.6, *p* < 0.001				
			Remission Remission at week 12 (post-treatment): 16/22 (73%)	Remission Remission at week 12 (post-treatment): 7/22 (32%) Between-group difference: 41%, *p* = 0.007				
			Remission at week 16: 11/19 (58%)	Remission at week 16: 2/19 (10%) Between-group difference: 48%, *p* = 0.002				

Abbreviations: C, control; CAPS, Clinician Administered PTSD Scale; DTS, Davidson Trauma Scale; I, intervention; IES-R, Impact of Event Scale-Revised; MMPI, Minnesota Multiphasic Personality Inventory; PTSD, post-traumatic stress disorder.

A negative between-group difference in change indicates a difference in favor of neurofeedback.

a + indicates no or minor problems; ? indicates some problems; and − indicates major problems.

**Figure 2.**
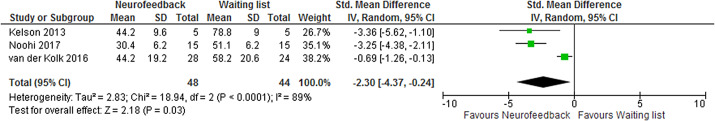
Meta-analysis of self-reported post-traumatic stress disorder symptoms after treatment with neurofeedback compared with waiting list.

Three studies [34-36] compared NF with no treatment and could be combined in meta-analysis. All three studies had very serious limitations and imprecision, for example, different preconditions in control treatment, unclear randomization, limitations in blinding, and/or small sample size. Different symptom scales were used to measure PTSD symptoms of which all but one, used in the study by Kelson [34], were validated. Treatment length ranged from 4 weeks to 12 weeks. All studies showed significant differences in favor of NF, both regarding the severity of symptoms and the number of patients achieving remission from PTSD (reported in two studies). The intervention groups showed a reduction in PTSD symptoms post-treatment of between 34 and 66%, compared with changes in the control groups ranging from a reduction of 14% to an increase of 13%. Meta-analysis of the pooled data shows a significant SMD of −2.30 (95% CI −4.37 to −0.24) post-treatment, but with very high heterogeneity (Figure 2).

Follow-up assessment was done in one of the studies [36]. At 1 month follow-up, a reduction of PTSD symptoms of 34% on the Davidson Trauma Scale was seen after NF versus 8% in the control group (*p* < 0.001).

One study [36] also measured symptom relief reported on the interview-based Clinician-Administered PTSD Scale. Post-treatment, a symptom reduction of 46% was seen in the NF group versus 13% in the waiting list group (*p* < 0.001). In this study, patients with treatment nonresponsive PTSD were included. Both groups continued ongoing treatment during the study period (medication and psychotherapy). At the 1-month follow-up, the symptom reduction from baseline was 51% in the NF group versus 14% in the waiting list group (*p* < 0.001).

The same study [36] also showed that remission 1-month post-treatment was achieved in 11/19 cases who had received NF treatment compared with 2/19 in the waiting list group (*p* = 0.002).

Based on our GRADE assessment (Table 2), we conclude that it is uncertain whether EEG-based NF compared with standard treatment or with waiting list reduces PTSD symptoms in adult patients with PTSD (very low certainty of evidence).

#### Level of functioning

One study [35], with very serious study limitations and imprecision, measured level of functioning using the Wisconsin Card Sorting Test and Tower of London. Both tests assess the ability to plan and adjust actions to stimuli and are validated scales for measuring executive cognitive functioning. Measures of both tests favored NF compared with the control group (*p* < 0.001), although no treatment is described for the comparison group in the publication (Table 4). Based on our GRADE assessment (Table 2), we conclude that it is uncertain whether EEG-based NF compared with no intervention improves the level of executive cognitive functioning in adult patients with PTSD (very low certainty of evidence).

**Table 4.** Reported effects on level of functioning

Study	Number of patients (*n*)	Withdrawal or dropout	Results		Comments	Directness^a^	Limitations^a^	Precision^a^
			EEG-based neurofeedback	No intervention				
Noohi, 2017 [35]	30	0	Wisconsin Card Sorting Test Number of errors Baseline: 75.7 (SD 22.7) After 45 days: 19.2 (SD 15.3) Δ −56.5	Wisconsin Card Sorting Test Number of errors Baseline: 73.7 (SD 21.4) After 45 days: 46.6 (SD 25.0) Δ −27.1 Between-group difference in pre-to-post change: Δ −29.4, *p* < 0.001	Both measures are well established cognitive performance tests that assess executive functioning. • Number of errors in WCST indicates rate of success in identifying a pattern. A lower score indicates a better result. • Perseveration response indicates how well an individual adjusts to a changed situation. A lower score indicates a better result. • Number of categories is a measure of how many tests an individual makes in the given time frame. In TOL a higher score indicates a better result. Both tests measure the actual number, which theoretically can be between 0 and infinity.	+	−	?
			Perseveration response Baseline: 49.8 (SD 18.0) After 45 days: 9.7 (SD 9.0) Δ −40.1	Perseveration response Baseline: 50.4 (SD 17.9) After 45 days: 26.5 (SD 18.5) Δ −23.9 Between-group difference in pre-to-post change: Δ −16.2, *p* < 0.001				
			Numbers of categories Baseline: 2.5 (SD 1.8) After 45 days: 5.6 (SD 0.7) Δ +3.1	Numbers of categories Baseline: 3.2 (SD 1.7) After 45 days: 4.2 (SD 1.7) Δ +1.0 Between-group difference in pre-to-post change: Δ +2.1, *p* < 0.001				
			*Tower of London* Score Baseline: 22.0 (SD 5.0) After 45 days: 28.1 (SD 3.2) Δ +6.1	*Tower of London* Score Baseline: 23.3 (SD 5.1) After 45 days: 23.3 (SD 3.1) Δ 0 Between-group difference in pre-to-post change: Δ +6.1, *p* < 0.001				

Abbreviations: TOL, Tower of London; WCST, Wisconsin Card Sorting Test.

a + indicates no or minor problems; ? indicates some problems; and − indicates major problems.

#### Medication use

Psychotropic medication use was evaluated in one study [25], with serious study limitations, indirectness, and serious imprecision. EEG-based NF was compared with standard treatment and psychotropic medication use was monitored by study physicians and reported after the study period. Two patients were not on medication at the study start. Of the 27 patients that were evaluated, all patients in the NF group (14/14) had reduced medication use according to a prespecified protocol as compared with 1/13 in the control group. The difference in proportion between groups was *χ* ^2^ = 23.26 (*p* < 0.05). Based on our GRADE assessment (Table 2), we conclude that it is uncertain whether EEG-based NF compared with other treatment reduces medication use in adult patients with PTSD (very low certainty of evidence).

#### Complications

In one study [34], participants were informed before the NF treatment about potential complications and continually asked to report any complications or side effects they may experience during or after the NF treatment, to either the clinician delivering the treatment or to the study coordinator. No participant reported any complications. In the study by van der Kolk et al. [36], one of 28 patients reported significant side effects after NF treatment, an increase in flashbacks. In the study by Noohi et al.[35], patients (number not stated) reported re-experiencing traumatic events and higher-than-normal levels of anxiety and stimulation. Although the risk of complications does not appear to be high, no firm conclusions can be drawn with regard to complications because data on complications were insufficiently collected and/or reported.

None of the included studies evaluated health-related quality of life, sick leave, or patients’ experiences of being treated with NF.

## Discussion

The findings of the four, small, studies included in this review, suggest that treatment with EEG-based NF may improve PTSD symptoms in adult patients with PTSD. When data from the individual studies were pooled in meta-analysis, the effect size shown was very large, SMD 2.3. Normally an SMD above 0.8 is considered a large effect size [37]. However, due to study limitations, some indirectness and imprecision, our confidence in this finding is very low. In addition, no sham-controlled study was identified, which implies that a placebo effect cannot be excluded. This means that we cannot draw any firm conclusions about the effects of the NF interventions. As to the other outcomes evaluated in the included studies, it is uncertain whether suicidal thoughts, executive functioning or medication use are affected, with certainty of evidence for these findings being very low.

All included studies show results in favor of EEG-NF. However, the findings are based on few and small studies with several study limitations, and there is also uncertainty with regard to directness (i.e., generalizability). Three of the four studies involved only men, while in the fourth, the majority were women. In two studies, the participants were war veterans; in one of them they were homeless war veterans in the United States. These factors limit the generalizability of the results to healthcare settings differing from those that are included in the studies. Although complications were not systematically addressed in the included studies, the rate seems to be low.

Despite the low confidence in the observed effect of NF, the promising findings from the included studies offer potential clinical implications. Most importantly, as clinicians report that PTSD treatments may halt in an initial stabilization phase, or even at the assessment, NF could be considered as an add-on to standard PTSD treatments. For example, in cases of treatment-resistant PTSD where patients are motivated to try NF, and in combination with clinicians’ professional judgments, we can see situations where NF can be applied. Possibly, these situations should be restricted to cases matching the samples in the studies included in our overview, but professionals ultimately must decide when to offer NF. Naturally, close follow-up by the professional would then be crucial, as would the necessity for patients to understand information on NF features as well as the state of research on the method. For policy makers, the findings in this review indicate the need for further research, and for cautious application of NF where relevant, while waiting for more robust research results.

The main limitations of this review are the small number of studies identified, and the small sample sizes and heterogeneity in terms of treatment protocols of the included studies. No study was identified that compared NF to sham NF, which limits the conclusions that can be drawn regarding the effectiveness of the intervention. Three of the studies compared NF with a nonactive waiting list and only one study compared with standard treatment. Besides small sample sizes, the included studies have several important limitations. One is the high drop-out rate, which might influence the results; however, it should be noted that this is a vulnerable patient group that may be predisposed to other factors interfering with possibilities of completing treatment. Furthermore, there was considerable variability in the intensity, dose, and character of NF treatment and protocols in the different studies; some delivered daily treatment and others less frequent sessions, the duration of treatment varied, and the number of sessions and type of NF differed among studies. EEG-NF was often provided in combination with mindfulness-related/meditation-related exercises. Finally, we did not perform a formal publication bias assessment using graphical or statistical methods, or did we perform any subgroup or sensitivity analyses, due to the small number of published studies. Taken together, these important limitations reduce the confidence we can place in the effect estimates.

The findings of this review are in agreement with three other reviews, which all interpret the evidence as limited, but indicate positive effects of NF in patients with PTSD [27,28,38]. Those reviews had slightly different inclusion criteria and therefore did not include the same four RCTs that were analyzed in this review, although there was some degree of overlap.

The effect size of the symptom reduction that was shown in the meta-analysis can be compared with two recent systematic reviews of other interventions for patients with PTSD [14, 15]. One showed an effect size for internet-based cognitive therapy compared with waiting list of SMD −0.60 (95% CI −0.97 to −0.24) [15], while the other showed an effect size for yoga, a method that often incorporates meditation-related exercises, compared with waiting list of SMD −1.10 (95% CI −1.72 to −0.47) [14]. In comparison with these reviews, the effect size for NF compares favorably. Our findings are also in line with the recently published NICE guideline on nonpharmacological interventions for adult patients with PTSD [39]. The guideline authors also conclude that there is low to very low evidence that NF results in large and statistically significant benefits in patients with PTSD on improving PTSD symptoms.

The findings of the review suggest a need for further intervention studies of EEG-based NF for patients with PTSD, especially in light of recent migration patterns of people who have been exposed to war trauma. Future studies should be rigorously designed and ideally compare NF with sham NF or an active treatment. A comparison of the number of sessions to symptom reduction for a stepwise analysis might give further indications of whether the reported effects can be attributed to the NF treatment. Moreover, to achieve detailed knowledge that can assist professionals’ decision making in individual PTSD treatments, studies on *when* (e.g., timing in relation to key life factors) and for *whom* (e.g., gender, age, stress levels, language, and culture background factors) the treatment would be beneficial, as well as studies that evaluate important outcomes such as suicidality, health-related quality of life, and complications. Furthermore, information on long-term effects of NF treatment for patients with PTSD is lacking. A number of issues need to be addressed in future studies such as; whether the observed effect is due to NF or to a placebo effect (extra effort and attention involved, that is, the extra number of sessions with a healthcare provider), whether the effect of NF is specific to the EEG feedback and not to meditation-related exercises, and whether there are less costly ways to perform NF (fewer sessions, at home etc.). EEG-based NF thus needs to be tested further in well-designed studies before being considered for evidence-based recommendation in clinical guidelines and thereby being incorporated in routine health care. Furthermore, studies that explore patients’ experiences of NF are also needed.

## Conclusion

In conclusion, our systematic review identified only four, small, RCTs, with several study limitations and imprecision, precluding us from drawing any strong conclusions about the effectiveness of EEG-based NF. Based on our GRADE assessment, we conclude that it is uncertain whether EEG-based NF compared with no treatment or standard treatment reduces PTSD symptoms post-treatment in adult patients with PTSD, as well as whether it results in any difference in suicidal thoughts, level of functioning, or medication use compared with no or other treatment (very low certainty of evidence). Given the need for effective treatment options for PTSD, further research on the use of EEG-based NF for this population is motivated.

## Supplementary material

For supplementary material accompanying this paper visit http://dx.doi.org/10.1192/j.eurpsy.2019.7. click here to view supplementary material
