# Supplementary material for: Electroencephalography-based neurofeedback as treatment for post-traumatic stress disorder: A systematic review and meta-analysis
Source: Eur Psychiatry. 2020 Jan 31;63(1):e7. doi: 10.1192/j.eurpsy.2019.7 (PMC8057448; doi:10.1192/j.eurpsy.2019.7)
Supplement: Supplementary file 1 [file S0924933819000075sup001.docx]

**Supplement**

Supplementary table 1. Excluded studies

| First author, year | Reason for exclusion |
| --- | --- |
| Banerjee, 2017 | Wrong publication type |
| Gapen, 2016 | Case series not reporting complications |
| Johnson, 2013 | Wrong population, not possible to separate PTSD |
| Kluetsch, 2014 | Wrong outcome |
| McReynolds, 2017 | Case series not reporting complications |
| Nicholson, 2016 | Case series not reporting complications |
| Panisch, 2018 | Wrong publication type |
| Peniston, 1993 | Case series not reporting complications |
| Reiter, 2016 | Wrong publication type |
| Ros, 2017 | Wrong population, healthy adults |
